# Age-related differences in sepsis outcomes: A comparative analysis of elderly and very elderly ICU patients

**DOI:** 10.2478/jccm-2025-0034

**Published:** 2025-10-31

**Authors:** Ozgur Kilic, Enver Demircan

**Affiliations:** Ondokuz Mayis University Faculty of Medicine, Department of Internal Medicine, Division of Intensive Care, Samsun, Turkey; Ondokuz Mayis University Faculty of Medicine, Department of Internal Medicine, Samsun, Turkey

**Keywords:** sepsis, elderly, very elderly, ICU outcomes, mortality predictors

## Abstract

**Background:**

The rapid aging of the global population has amplified the clinical and economic burden of sepsis, a leading cause of morbidity and mortality in the elderly. Within this demographic, the “very elderly” (≥80 years) represent a particularly vulnerable subgroup. This study evaluates and compares the outcomes and prognostic factors of elderly (65–79 years) and very elderly ICU patients with sepsis or septic shock.

**Methods:**

A retrospective observational study was conducted in a single-center ICU, including 251 patients aged ≥65 years diagnosed with sepsis or septic shock. Patients were categorized as elderly (65–79 years, N=162) or very elderly (≥80 years, N=89). Data on demographics, comorbidities, laboratory results, infection sources, treatments, and outcomes were collected. Prognostic factors for mortality were analyzed using binary logistic regression.

**Results:**

The very elderly group exhibited higher rates of dementia, immobility, and fungal infections, while malignancy was more prevalent in the elderly group. ICU length of stay was longer in the very elderly group (median 8 vs. 6 days, P=0.027). ICU mortality was lower in the very elderly group, showing a trend toward significance but not reaching statistical significance (70.8% vs. 82.1%, P=0.056). Shared predictors of mortality included higher SOFA scores, malignancy, hospital-acquired sepsis, invasive mechanical ventilation, and acute kidney injury.

**Conclusion:**

This study highlights differences in sepsis outcomes between elderly and very elderly patients. The findings underscore the importance of developing and implementing age-specific management strategies to improve outcomes in these high-risk populations. These insights contribute to a more tailored and effective approach to geriatric critical care.

## Introduction

The global trend toward an aging population—often described as the “silver tsunami”—has profound implications for healthcare systems [1]. Advances in medical care and public health have extended life expectancy, leading to a growing number of individuals aged 65 and above. Among them, the “very elderly” (≥80 years) represent a particularly fragile subgroup due to their increased vulnerability to chronic conditions and frailty [2].

Sepsis, a life-threatening syndrome driven by a dysregulated immune response to infection, is especially prevalent and deadly in older adults [3]. Age-related physiological changes, such as immunosenescence, multiple comorbidities, and diminished organ reserve, increase susceptibility and complicate clinical management [4]. Immunosenescence, characterized by a gradual decline in both innate and adaptive immune responses, reduces the ability to effectively recognize and clear pathogens. This leads to a delayed and often inadequate immune reaction, contributing to higher infection rates and poorer outcomes in the very elderly [5]. The very elderly often present additional challenges, including altered pharmacokinetics and functional decline, which may influence sepsis progression and treatment outcomes [4, 5].

Despite its clinical significance, comparative data on sepsis outcomes between elderly (65–79 years) and very elderly (≥80 years) patients remain scarce. Understanding how these groups differ in risk profiles and outcomes is essential for optimizing ICU care and resource allocation.

This single-center retrospective study aims to bridge this gap by examining the clinical characteristics and outcomes of elderly versus very elderly patients admitted to the ICU with sepsis or septic shock. The findings aim to inform age-specific strategies for sepsis management and contribute to the broader discourse on geriatric critical care.

## Materials and Methods

### Study Design and Setting

This retrospective observational study was conducted in the Internal Medicine Intensive Care Units (ICUs) of Ondokuz Mayıs University between June 2022 and June 2023. The study was designed to evaluate the prognosis of elderly and very elderly patients diagnosed with sepsis or septic shock. Ethical approval was obtained from the Ondokuz Mayıs University Ethics Committee (Approval Date: December 4, 2023, Approval Number: 2023/363).

### Patient Selection

Patients aged 65 and older, diagnosed with sepsis or septic shock prior to or during their ICU stay, were enrolled in the study. They were categorized into two age groups: elderly (65–79 years) and very elderly (80 years and above). Inclusion criteria required admission to the medical ICUs during the study period, meeting the diagnostic criteria for sepsis or septic shock as outlined by the Sepsis-3 consensus definitions, and being aged 65 years or older. Exclusion criteria included patients younger than 65 years, those in the terminal stages of disease, and those with ICU stays of less than 24 hours.

### Diagnosis of Sepsis

Sepsis was defined based on the Sepsis-3 criteria, which characterize it as a life-threatening condition resulting from organ dysfunction caused by a dysregulated host response to infection. The degree of organ dysfunction was assessed using the Sequential Organ Failure Assessment (SOFA) score, with a two-point or greater increase from baseline indicating sepsis. Septic shock was identified in patients requiring vasopressor support to sustain a mean arterial pressure of ≥65 mmHg, accompanied by serum lactate levels above 2 mmol/L despite adequate fluid resuscitation [6]. Clinical indicators such as fever, tachycardia, tachypnea, and leukocytosis or leukopenia were considered alongside biomarkers including procalcitonin and C-reactive protein. Blood cultures were obtained before initiating antimicrobial therapy to identify causative pathogens, supplemented by other microbiological investigations, including urine, sputum, and wound cultures when indicated. Imaging modalities, such as chest X-rays and abdominal ultrasonography, were employed to localize infection sources, with computed tomography used for suspected abdominal foci. The qSOFA score served as a quick screening tool to identify high-risk patients outside the ICU. It relies on three clinical parameters: changes in mental status, a respiratory rate of 22 or more breaths per minute, and a systolic blood pressure of 100 mmHg or lower [6].

### Etiology of Sepsis

The pathogens responsible for sepsis were categorized into four groups: bacterial (further divided into Gram-positive and Gram-negative), fungal, and viral infections. The distribution of pathogens was evaluated in both age groups to identify significant differences between them.

### Source of Sepsis

The primary infection sites leading to sepsis were categorized as pulmonary, urinary, intra-abdominal, bloodstream, soft tissue, or central nervous system (CNS). These sources were compared between the two age groups to assess potential variances in infection localization.

### Origin of Sepsis

The origin of sepsis was categorized as either community-acquired or hospital-acquired, based on the timing of infection diagnosis in relation to hospital admission. Community-acquired sepsis was defined as an infection diagnosed within 48 hours of admission, while hospital-acquired sepsis referred to infections diagnosed after 48 hours of admission or in patients with recent hospitalization or healthcare exposure within the past 90 days, following established definitions [7].

### Data Collection

Patient data were retrieved retrospectively from ICU records and hospital databases. Collected parameters included demographic characteristics such as age, gender, and comorbidities, including malignancy. Laboratory data comprised laboratory results, including CRP, procalcitonin, WBC, hemoglobin, platelets, creatinine, glucose, pH, pCO_2_, and lactate levels. Disease severity scores, including the SOFA and APACHE II scores, were calculated to assess organ dysfunction and predict prognosis. The Charlson Comorbidity Index (CCI) was calculated to quantify the burden of chronic diseases for each patient. The CCI assigns weighted scores to various comorbidities based on their potential impact on mortality, with higher scores indicating greater comorbidity burden. Conditions such as malignancy, cardiovascular disease, and renal failure contribute to the index, which is widely used in critical care research to stratify patient risk [8]. Treatment modalities such as the use of vasopressors, invasive mechanical ventilation, and hemodialysis were recorded. The study outcomes included invasive mechanical ventilation length of stay (IMV LOS), intensive care unit length of stay (ICU LOS), and intensive care unit mortality (ICU mortality). Acute kidney injury (AKI) was also recorded as a potential factor influencing mortality.

### Statistical Analysis

The data were analyzed using SPSS version 21 (IBM Corp., NY, USA). Descriptive statistics were used to summarize demographic and clinical data, with continuous variables presented as means, medians, ranges (minimums and maximums), standard deviations, and interquartile ranges. The Shapiro-Wilk and Kolmogorov-Smirnov tests were used to check whether the data followed a normal distribution. For data with a normal distribution, the Independent Samples t-test was used for group comparisons, while the Mann-Whitney U test was applied to non-normally distributed data. Binary logistic regression was performed to examine the effects of independent variables on binary outcomes, with results reported as odds ratios and 95% confidence intervals. A p-value of less than 0.05 was considered statistically significant.

## Results

The study included 251 patients, divided into two groups: elderly patients aged 65–79 years (N=162) and very elderly patients aged 80+ years (N=89). The gender distribution was comparable between the groups, with no statistically significant difference (P=0.351) (Table 1). The very elderly group exhibited a significantly higher prevalence of dementia (25.8% vs. 6.8%, P<0.001) and immobility (11.2% vs. 2.5%, P=0.007). Conversely, the elderly group had a higher rate of malignancy (61.1% vs. 27.0%, P<0.001). The Charlson Comorbidity Index was notably higher in the elderly group (mean 4.1±2.3 vs. 3.2±2.1, P<0.001) (Table 1).

**Table 1.** Comparison of Clinical, Laboratory, and Outcome Parameters in Elderly (65–79 Years) and Very Elderly (80+ Years) Septic Patients

**Parameters**	**65–79 Years, N=162**	**80+ Years, N=89**	**P value**
Gender, N (%)			
Male	99 (61.1)	49 (55.1)	0.351
Female	63 (38.9)	40 (44.9)	

Comorbidities, N (%)			
Diabetes Mellitus	63 (38.9)	27 (30.3)	0.177
Hypertension	66 (40.7)	28 (31.5)	0.146
Chronic kidney disease	23 (14.2)	22 (24.7)	0.057
End stage kidney disease	7 (4.3)	2 (2.2)	0.498
Chronic pulmonary disease	20 (12.3)	15 (16.9)	0.426
Congestive heart failure	27 (16.7)	22 (24.7)	0.17
Coronary artery disease	47 (29.0)	28 (31.5)	0.685
Cerebrovascular disease	11 (6.8)	16 (18.0)	0.012
İmmobility	4 (2.5)	10 (11.2)	0.007
Dementia	11 (6.8)	23 (25.8)	<0.001
Liver disease	10 (6.2)	6 (6.7)	0.893
Rheumatic disease	8 (4.9)	1 (1.1)	0.165
Malignancy	99 (61.1)	24 (27.0)	< 0.001
Chalrlson Comorbidity Index, mean (SD)	4.10 (2.30)	3.2 (2.10)	<0.001

Laboratory, median (IQR)			
CRP, mg/L	141.53 (87.02–209.54)	125.00 (83.52–195.54)	0.179
Procalcitonin, ng/mL	2.94 (0.62–16.03)	2.40 (0.52–8.01)	0.180
WBC, 1000/µL	12.33 (7.60–17.71)	13.02 (10.04–18.01)	0.280
Hb, g/Ll	9.42 (8.43–10.90)	10.03 (9.04–11.02)	0.009
PLT, 1000/µL	149.13 (70.53–246.54)	200.34 (110.21–293.40)	0.011
Crea, mg/dL	1.81 (0.94–2.91)	1.63 (1.12–2.92)	0.599
PH	7.38 (7.30–7.44)	7.39 (7.28–7.45)	0.487
PCO2, mmHg	40.03 (34.02–49.31)	40.04 (34.03–54.01)	0.585
Lactate, mmol/L	2.11 (1.42–3.30)	2.04 (1.51–3.11)	0.859

Organ Support, N (%)			
IMV	72 (44.4)	55 (61.0)	0.009
Vasopressor/Inotrope	127 (78.4)	76 (85.4)	0.238
HD	46 (28.4)	20 (22.5)	0.308

Severity of disease, mean (SD) point			
SOFA	10.50 (3.90)	9.80 (3.20)	0.149
APACHE II	25.80 (8.10)	25.20 (7.60)	0.863

Outcomes			
IMV LOS, median (IQR) day	2.00 (0.00–7.00)	4.00 (1.00–9.00)	0.090
ICU LOS, median (IQR) day	6.00 (3.00–11.00	8.00 (4.00–13.00)	0.027
ICU mortality, N (%)	133 (82.1)	63 (70.8)	0.056

**Abbreviations:** N (%) : Number (percentage), CRP : C-reactive protein, WBC : White blood cell count, Hb : Hemoglobin, PLT : Platelet count, Crea : Creatinine, PH : Blood pH level, PCO2 : Partial pressure of carbon dioxide, IMV : Invasive mechanical ventilation, HD : Hemodialysis, SOFA : Sequential Organ Failure Assessment score, APACHE II : Acute Physiology and Chronic Health Evaluation II score, LOS : Length of stay, ICU : Intensive care unit, SD : Standard deviation, IQR : Interquartile range, P value : Probability value indicating statistical significance.

Laboratory parameters, including CRP, procalcitonin, and lactate levels, did not differ significantly between the groups. However, hemoglobin levels were higher in the very elderly group (mean 10.0±1.0 g/L vs. 9.4±1.0 g/L, P=0.009), and platelet counts were also significantly elevated in this group (200×10^3^/µL vs. 149×10^3^/µL, P=0.011) (Table 1).

The need for invasive mechanical ventilation (IMV) was significantly higher in the very elderly group (61.0% vs. 44.4%, P=0.009). Vasopressor/inotrope and hemodialysis support needs were comparable between groups. Disease severity scores, including SOFA and APACHE II, were not statistically different between groups (Table 1).

**Fig. 1.**
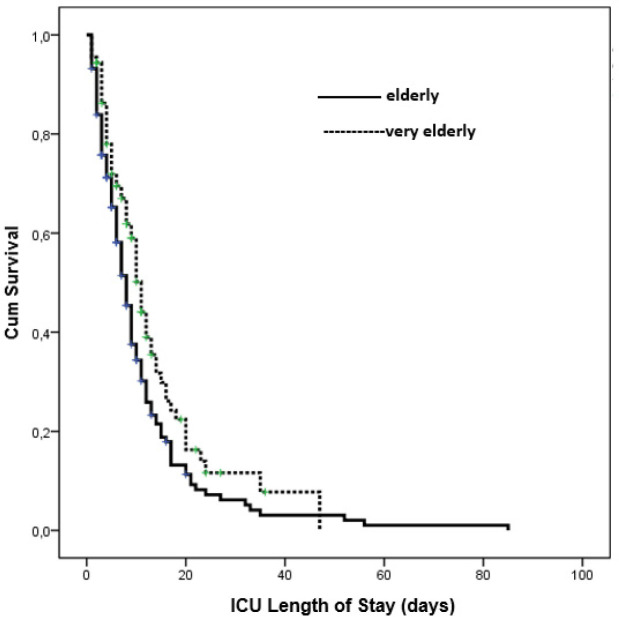
Kaplan–Meier survival curve comparing ICU mortality between elderly (65–79 years) and very elderly (80+ years) sepsis patients.

The median duration of invasive mechanical ventilation (IMV) was numerically longer in the very elderly group compared to the elderly group (4 days [IQR: 1–9] vs. 2 days [IQR: 0–7]); however, this difference did not reach statistical significance (P=0.090). In contrast, the median ICU length of stay (LOS) was significantly longer in the very elderly group (8 days [IQR: 4–13]) compared to the elderly group (6 days [IQR: 3–11], P=0.027). Although ICU mortality was lower in the very elderly group (70.8% vs. 82.1%), the difference was not statistically significant (P=0.056) (Table 1,Figure 1).

Fungal infections were more prevalent in the very elderly group (34.8% vs. 21.0%, P=0.017) (Table 2). Pulmonary infections were the most common sepsis source for both groups, with no significant difference (P=0.481). However, urinary infections trended higher in the very elderly (21.3% vs. 13.0%, P=0.083) (Table 2).

**Table 2.** Comparison of Sepsis Etiology, Sources, and Origins by Age Group

**Etiology of sepsis**	**65–79 Years old, N (%)**	**80 Years and older N (%)**	**p Value**
Bacterial infection			
Gram-positive	92 (56.8)	46 (51.7)	0.437
Gram-negative	67 (41.4)	43 (48.3)	0.288
Fungal infection	34 (21.0)	31 (34.8)	0.017
Viral infection	1 (0.6)	0 (0)	0.458

Source of sepsis			
Pulmonary	89 (54.9)	53 (59.6)	0.481
Urinary	21 (13.0)	19 (21.3)	0.083
Intra-abdominal	47 (29.0)	20 (22.5)	0.262
Bloodstream	10 (6.2)	8 (9.0)	0.408
Soft tissue	7 (4.3)	4 (4.5)	0.949
Central nervous system	6 (3.7)	0 (0)	0.066

Origin of sepsis			0.329
Community	59 (36.4)	38 (42.7)	
Hospital	103 (63.6)	51 (57.3)	

In the univariate analysis, several factors were significantly associated with ICU mortality in both groups. In the elderly group, higher SOFA and APACHE II scores, elevated lactate levels, malignancy, need for IMV or vasopressor support, fungal infection, hospital-acquired sepsis, and acute kidney injury were significantly related to increased mortality (Table 3). Similarly, in the very elderly group, univariate predictors of mortality included higher SOFA and APACHE II scores, elevated PCO_2_, malignancy, IMV, vasopressor use, fungal infection, hospital-acquired sepsis, and acute kidney injury (Table 4).

**Table 3.** Factors Predicting Mortality in Elderly Patients with Sepsis

**Factor**	**Univariate OR (95% CI)**	P *	**Multivariate OR (95% CI**	P *
Severity of disease				
SOFA	1.23 (1.17,1.42)	<0.001	1.26 (1.07, 1.48)	0.005
APACHE	1.11 (1.06,1.15)	<0.001		

Laboratory				
Lactate, mmol/L	1.28 (1.04, 1.59)	0.022		

Comorbidity				
Malignancy	1.46 (1.11, 1.92)	0.007	1.38 (1.04, 2.10)	0.039

Organ support				
IMV	2.26 (1.59, 3.20)	<0.001		
Vasopressor/Inotrope	1.82 (1.31, 2.50)	<0.001		

Etiology of sepsis				
Fungal infection	1.67 (1.12, 2.47)	0.011	1.53 (1.01, 2.33)	0.046

Origin of sepsis				
Hospital acquired	2.12 (1.51, 2.97)	<0.001	1.74 (1.02, 3.08)	0.019

Events				
Acute kidney injury	2.39 (1.72, 3.31)	<0.001	2.18 (1.29, 3.72)	0.003

* Only variables with p < 0.05 are presented in the table. Variables with non-significant p-values or wide confidence intervals were excluded from the table due to their lack of clear association with mortality. These factors were considered in the full model but are not reported here to maintain focus on significant findings.

**Table 4.** Factors Predicting Mortality in Very Elderly Patients with Sepsis

**Factor**	**Univariate OR (95% CI)**	P *	**Multivariate OR (95% CI**	P *
Severity of disease				
SOFA	1.22 (1.05, 1.42)	0.006	1.24 (1.04–1.49)	0.017
APACHE	1.10 (1.04, 1.18)	<0.001		

Laboratory				
PCO2, mmHg	1.03 (1.01, 1.06)	0.021		

Comorbidity				
Malignancy	2.14 (1.15, 3.99)	0.016	1.63 (1.02–2.46)	0.026

Organ support				
IMV	2.14 (1.41, 3.25)	<0.001		
Vasopressor/Inotrope	1.66 (1.20, 2.30)	0.002		

Etiology of sepsis				
Fungal infection	1.59 (1.03, 2.44)	0.034	1.45 (1.01–2.08)	0.045

Origin of sepsis				
Hospital acquired	2.01 (1.34, 3.00)	<0.001	1.88 (1.11–3.20)	0.022

Events				
Acute kidney injury	2.28 (1.30, 4.00)	0.003	2.12 (1.18–3.79)	0.007

* Only variables with p < 0.05 are presented in the table. Variables with non-significant p-values or wide confidence intervals were excluded from the table due to their lack of clear association with mortality. These factors were considered in the full model but are not reported here to maintain focus on significant findings.

Mortality predictors identified through multivariate analysis revealed both shared and distinct factors between the elderly (65–79 years) and very elderly (≥80 years) groups. In the elderly group, higher SOFA scores significantly predicted mortality (OR: 1.26, 95% CI: 1.07–1.48, P=0.005). Elevated lactate levels were also associated with increased mortality risk (OR: 1.28, 95% CI: 1.04–1.59, P=0.022). Among comorbidities, malignancy was a significant risk factor (OR: 1.38, 95% CI: 1.04–2.10, P=0.039). The need for invasive mechanical ventilation (IMV) markedly increased the risk of mortality (OR: 2.26, 95% CI: 1.59–3.20, P<0.001), as did vasopressor/inotrope use (OR: 1.82, 95% CI: 1.31–2.50, P<0.001). Fungal infections emerged as a significant etiological predictor (OR: 1.53, 95% CI: 1.01–2.33, P=0.046), and hospital-acquired sepsis also significantly heightened mortality risk (OR: 1.74, 95% CI: 1.02–3.08, P=0.019). Additionally, acute kidney injury substantially increased mortality (OR: 2.18, 95% CI: 1.29–3.72, P=0.003) (Table 3).

In the very elderly group, several predictors were consistent with the elderly group, while others were unique. Higher SOFA scores were associated with mortality (OR: 1.24, 95% CI: 1.04–1.49, P=0.017), and PCO_2_ levels were also significant (OR: 1.03, 95% CI: 1.01–1.06, P=0.021). Malignancy remained a key risk factor (OR: 1.63, 95% CI: 1.02–2.46, P=0.026), as did IMV (OR: 2.14, 95% CI: 1.41–3.25, P<0.001) and vasopressor/inotrope use (OR: 1.66, 95% CI: 1.20–2.30, P=0.002). Fungal infections (OR: 1.45, 95% CI: 1.01–2.08, P=0.045) and hospital-acquired sepsis (OR: 1.88, 95% CI: 1.11–3.20, P=0.022) were also significant predictors. Acute kidney injury again showed a strong association with mortality (OR: 2.12, 95% CI: 1.18–3.79, P=0.007) (Table 4).

## Discussion

This study provides important insights into the outcomes and clinical characteristics of elderly (65–79 years) and very elderly (≥80 years) patients with sepsis or septic shock. Despite longer ICU stays, the very elderly group exhibited lower, though statistically non-significant, mortality—a pattern that challenges conventional assumptions about aging and sepsis outcomes. One plausible explanation is survivor bias, as individuals who reach advanced age may possess greater physiological resilience [9,10,11]. Additionally, treatment decisions in this group often prioritize supportive care and are shaped by frailty, comorbidities, and patient-centered goals. Such individualized approaches may reduce treatment-related harm and better align with quality-of-life considerations [ 12, 13,14]. Finally, the absence of detailed frailty metrics in this study may have led to underestimating their contribution to outcomes.

The prolonged ICU length of stay observed in very elderly patients likely reflects both the clinical complexity associated with multimorbidity and the slower physiological recovery due to diminished organ reserve. In this population, cautious management strategies aimed at minimizing risks related to frailty, along with delays in discharge planning due to safety concerns and limited availability of appropriate post-ICU care, may further contribute to extended ICU occupancy [15, 16]. Whether such prolonged stays lead to improved long-term outcomes or merely reflect the challenges of critical care in this age group remains uncertain. Future research should examine this issue in greater depth, with particular attention to the integration of frailty and functional status assessments into care planning.

Malignancy was an independent predictor of ICU mortality in both elderly and very elderly patients with sepsis, consistent with prior studies linking cancer to poor outcomes [ 17, 18, 19, 20, 21, 22]. Immunosuppression from both the malignancy and its treatments impairs immune defenses, increasing susceptibility to infections and delaying pathogen clearance [ 18, 20, 21]. In the very elderly, this vulnerability may be amplified by frailty, comorbidities, and cumulative physiological decline [19]. Cancer-related complications such as neutropenia and catheter-associated infections often serve as sepsis triggers in this group [18, 22]. These findings highlight the importance of early sepsis recognition and tailored treatment strategies for patients with active malignancy to improve outcomes.

Comorbidities such as cardiovascular disease, dementia, and immobility were more prevalent in the very elderly and likely contributed to increased clinical vulnerability, even though they did not independently predict mortality. This suggests that the cumulative burden of comorbid conditions may be more impactful than individual diagnoses. While dementia and immobility are recognized frailty markers [23], their limited prognostic value in this study may reflect the dominant role of acute organ dysfunction during sepsis. Given the complex, multidimensional nature of frailty, integrating standardized frailty assessments into sepsis risk models may enhance prognostic accuracy and guide more personalized care in geriatric ICU populations [24, 25].

While lactate levels were associated with mortality in univariate analysis, this relationship did not remain significant in multivariate models, suggesting that other clinical factors had a stronger influence. Although lactate is widely used as a marker of tissue hypoperfusion, its interpretation in elderly patients is complicated by age-related physiological changes, such as altered metabolism, reduced hepatic clearance, and the presence of comorbidities like chronic kidney disease or diabetes [26]. These factors can elevate lactate levels independent of perfusion status. In this context, traditional biomarkers may be less reliable, and frailty or overall disease burden might offer more prognostic insight [27]. Therefore, lactate should be considered within a broader clinical framework when assessing outcomes in critically ill geriatric patients.

Severity scores such as SOFA and APACHE II showed predictive value for mortality in both elderly and very elderly patients, with SOFA remaining significant across age groups. While these scores effectively reflect organ dysfunction—a key determinant of sepsis outcomes—they may not fully account for physiological reserve or frailty [28]. The observation that very elderly patients experienced longer ICU stays and more frequent use of mechanical ventilation despite similar severity scores suggests that traditional models may underestimate vulnerability in this population. Incorporating frailty assessments alongside severity scoring could enhance prognostic accuracy and guide more individualized care strategies for older ICU patients [29].

Hospital-acquired sepsis was a strong predictor of mortality in both age groups, underscoring the clinical burden of nosocomial infections in critical care. These infections, often driven by multidrug-resistant organisms and prolonged exposure to invasive devices, pose a particular threat to older adults with compromised immunity [30]. These findings highlight the importance of robust infection prevention protocols and antimicrobial stewardship, especially in geriatric ICU settings. Fungal infections were significantly more common in the very elderly and independently associated with ICU mortality. This underscores their heightened vulnerability to opportunistic pathogens, driven by immunosenescence, broad-spectrum antibiotic use, and invasive procedures. Early recognition and timely antifungal therapy are essential, especially given the diagnostic delays and poor outcomes often seen in this population [31, 32].

Invasive mechanical ventilation (IMV) was independently associated with ICU mortality in the very elderly, but not in the younger elderly cohort. This disparity may reflect the diminished physiological reserve, frailty, and increased susceptibility to complications such as ventilator-associated pneumonia, barotrauma, and prolonged ICU stays in the oldest patients [24, 33]. Recovery from both the acute illness and the stress of mechanical ventilation is more challenging in this group, especially when compounded by comorbidities. In contrast, younger elderly patients may better tolerate IMV or may have been more selectively chosen for it [33]. These findings underscore the importance of individualized, risk-sensitive decision-making regarding IMV in very elderly patients, with attention to clinical benefit, patient preferences, and long-term outcomes.

Acute kidney injury (AKI) was a strong and independent predictor of ICU mortality in both elderly and very elderly patients. In the setting of sepsis, AKI signifies systemic inflammation, hypoperfusion, and nephrotoxic insult, all of which contribute to multi-organ failure [34]. Its impact is particularly pronounced in the very elderly due to diminished renal reserve, frailty, and comorbidities. Even mild AKI has been linked to significantly worse outcomes, highlighting the need for early recognition, careful fluid management, avoidance of nephrotoxins, and timely initiation of renal support [35]. These findings emphasize the importance of pro-active AKI management to improve survival in this high-risk population.

This study has some limitations. Its retrospective design made it difficult to fully evaluate frailty, a complex factor known to play a key role in sepsis outcomes. As a result, frailty’s impact on the findings may have been underestimated. Additionally, being a single-center study limits the ability to apply these results to other settings, as ICU practices and patient characteristics can vary widely.

This study, despite its limitations, adds to the growing body of knowledge on geriatric sepsis care by identifying key differences in outcomes and risk factors between elderly and very elderly patients. It emphasizes the importance of age-specific sepsis management approaches and highlights the need for future research addressing frailty assessments and tailored interventions. As the global population ages, these findings are critical for optimizing care and resource allocation for vulnerable ICU patients.

## Conclusion

This study reveals that sepsis affects elderly and very elderly ICU patients in clinically distinct ways, with age alone failing to capture the complexity of risk. Despite similar disease severity, the very elderly faced longer ICU stays and unique vulnerabilities—often shaped by frailty rather than classic markers. Key predictors of mortality, including organ dysfunction, malignancy, hospital-acquired and fungal infections, underscore the need for more nuanced, individualized approaches. Future strategies must go beyond age and integrate frailty and functional status to guide critical care in this growing population.
